# Case Report: Successful R0 resection in locally advanced retroperitoneal sarcomas

**DOI:** 10.3389/fsurg.2024.1343014

**Published:** 2024-01-22

**Authors:** Peter Bael, Bayan Alqtishat, Khaled Alshawwa

**Affiliations:** ^1^ Medical Research Club, Faculty of Medicine, Al-Quds University, Jerusalem, Palestine; ^2^ Department of General Surgery, Al-Makassed Charitable Society Hospital, Jerusalem, Palestine

**Keywords:** retroperitoneal sarcomas, IVC graft, R0 resection, liposarcoma, leiomyosarcoma, multidisciplinary involvement, case report

## Abstract

We present a case series of three successfully resected tumors in our center at Al-Makassed Hospital in Jerusalem, Palestine, all of which primarily involved or invaded adjacent structures and needed a multidisciplinary approach to achieve R0 resection. Our first patient is a 42-year-old previously healthy female with intermittent attacks of dull aching abdominal pain. Her tumor was a leiomyosarcoma that involved major vessels and other adjacent vital structures. Ultimately, she needed major highly advanced surgery necessitating the need for vascular reconstruction of the IVC, as well as R0 resection. The surgery was performed by a multidisciplinary team of highly specialized surgeons in related fields. Our second case is a 75-year-old female patient with a well-differentiated liposarcoma invading the upper pole of the right kidney, necessitating a nephrectomy. Consequently, this case demanded the interdisciplinary involvement of nephrology. Our third patient is a 59-year-old male with dedifferentiated liposarcoma that involved the spleen, pancreas, and splenic flexure while engulfing the left kidney and ureter. Beyond the removal of the tumor, multiorgan resection was imperative to achieve microscopic margin-free resection. This extensive local spread needed broad collaboration from the medical team and other surgical subspecialties. All surgeries went well, and their outcomes were promising. All patients had an uneventful follow-up and, to date, no recurrence. Invasive retroperitoneal sarcomas of different histological types and clinical stages represent a technical challenge. Careful preoperative investigation and an experienced, dedicated multidisciplinary team of surgeons and non-surgeons from related fields, including vascular, urologic, and hepatobiliary surgeons, are usually needed for a safe and successful R0 resection despite extensive tumor involvement in light of difficulty achieving early diagnosis.

## 1 Introduction

Retroperitoneal malignancies are rarer than their extremity counterparts and are among the rarest soft-tissue malignancies, representing only 12%–15% (1, 2). Their placement in the retroperitoneum makes their presentation vague, their diagnosis hard, and their excision tricky (1). Surgical management, though it is first-line treatment, is impeded and complicated when the tumor involves surrounding structures and vasculature, generating a high recurrence rate due to incomplete excision (1, 3).

This is especially true in the case of retroperitoneal liposarcoma, which calls for specialist management in centers with experience in management, and a multi-disciplinary team integrating surgery, radiology, and oncology, along with case-specific involvement from other fields. Diagnostic imaging is complex requiring multiple modalities, and post-operative follow-up is exhausting necessitating consistent imaging to detect recurrence (4).

R0 resection is defined as resection resulting in microscopically and macroscopically margin-negative resection, whereas R1 resection is restricted to macroscopic remission. Classically, R1 resection has been the mainstay of surgical treatment for retroperitoneal sarcomas, except in cases of invasion. Recent studies have challenged this concept and suggested compartmentalization as a method to decrease tumor recurrence, which, in the case of retroperitoneal sarcomas, is the leading cause of death (2). R0 excision has now become the gold standard of management of retroperitoneal sarcomas, with consistent improvement in survival rates (3, 4).

In our case series, we present multiple large sarcomas involving different, multiple, and obscure organs, causing various changes in their presentations and requiring multidisciplinary management. Nevertheless, in the end, they were successfully resected with R0 resection and have yet to recur.

Our first case is of a 42-year-old female whose leiomyosarcoma involved the inferior vena cava, ultimately demanding vascular reconstruction, and yet was managed successfully, with an uneventful postoperative course. The second case is of a 75-year-old female with liposarcoma invading the right kidney and thus requiring nephrectomy, with close renal observation postoperatively.

Our third and final case is of a 59-year-old male, whose dedifferentiated liposarcoma arose from the mesentery, which occurs in 2% of cases and involved varied structures, requiring multiorgan excision.

## 2 Case presentation

### 2.1 Case 1

**Figure 1:**
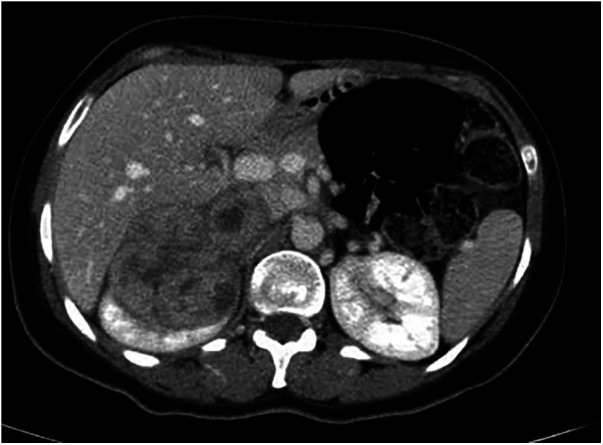
Ct, CT findings for case 1.

A 42-year-old female patient presented to our department complaining of intermittent attacks of dull aching abdominal pain localized at the top and upper right side of her abdomen. On examination, she had right upper quadrant tenderness but no palpable masses. An abdominal ultrasound revealed a heterogeneous lesion at the upper pole of her right kidney. Computerized tomography (CT) showed a heterogeneous lesion at the upper pole of the right kidney that measured 7*:*5 × 7 cm, invading adjacent liver parenchyma without clear cleavage (Figure 1—CT).

Abdominal MRI was performed next and showed a heterogeneously enhanced soft tissue mass in the upper pole of the right kidney measuring 6*.*8 × 9*.*5 cm, mainly involving segment VII of the liver and a small part of segment VIII. The mass abutted the inferior vena cava (IVC) and severely narrowed it, causing it to appear slit-like. The mass was circumferentially compressing the vein and surrounding it, and even stimulated a chronic inflammatory reaction with focal mural thrombosis, though it didn't seem to invade or penetrate the inferior vena cava itself. Furthermore, the mass seemed to involve the lower wall of the IVC. An ultrasound-guided tru-cut biopsy showed evidence of leiomyosarcoma with many mitotic figures.

Due to the complexity of the mass anatomy, a multidisciplinary team was formed, involving oncologists, surgeons, radiologists, and pathologists, who opted for surgical management aiming for R0 resection of the tumor, which involved drastic vascular, hepatic, and renal resection, and significant risk of morbidity and mortality. The combined efforts of all involved parties was essential, especially considering the high risk of bleeding, surrounding organ injury, and the importance of achieving negative margins.

Surgery was performed through the modified Makuuchi incision, in the supine position. Once the underlying tissue was separated to achieve access to the peritoneal cavity, we ligated and excised the falciform ligament. We divided the hepatocolic ligament, and carefully dissected any remaining attachments to the retroperitoneum, diaphragm, and IVC, allowing us to freely mobilize the liver. We kocherized and dissected the duodenum until the IVC was exposed and isolated. We could then visualize a large solid retroperitoneal mass, circumferentially engulfing the IVC to the level of the left renal vein and extending upwards to the hepatic veins while compressing the right liver lobe (segment VII). We dissected, separated, and isolated the IVC segment from the insertion of the hepatic vein to the level of the left renal vein. Subsequently, we performed the Pringle's maneuver for 5 min. The mass extension into segment VII of the liver was resected. The remaining parenchyma showed no involvement or abnormal changes. We then placed two stitches to secure hemostasis.

We then attempted to carefully separate the mass surrounding the IVC by fine sharp dissection; however, we were only partially successful due to the apparent involvement of the IVC wall. The left renal vein was isolated and controlled via a vessel loop. We applied two vascular clamps, one inferior to the left renal vein, and one superior to the right suprarenal vein. The right gonadal vein was dilated, though we managed to isolate and protect it as well. We applied three staples to the renal pedicle and performed a nephrectomy. We then clamped the right ureter.

A 10-cm segment of the IVC was obliquely resected along with the mass, up to the origin of the left renal vein. We then reconstructed the IVC by implementing a 22 mm Dacron graft, first sutured at the level of the liver hilum, and then above the renal vein. This was when we released the vascular clamps, with no detectable leakage, and we secured hemostasis.

**Figure 2:**
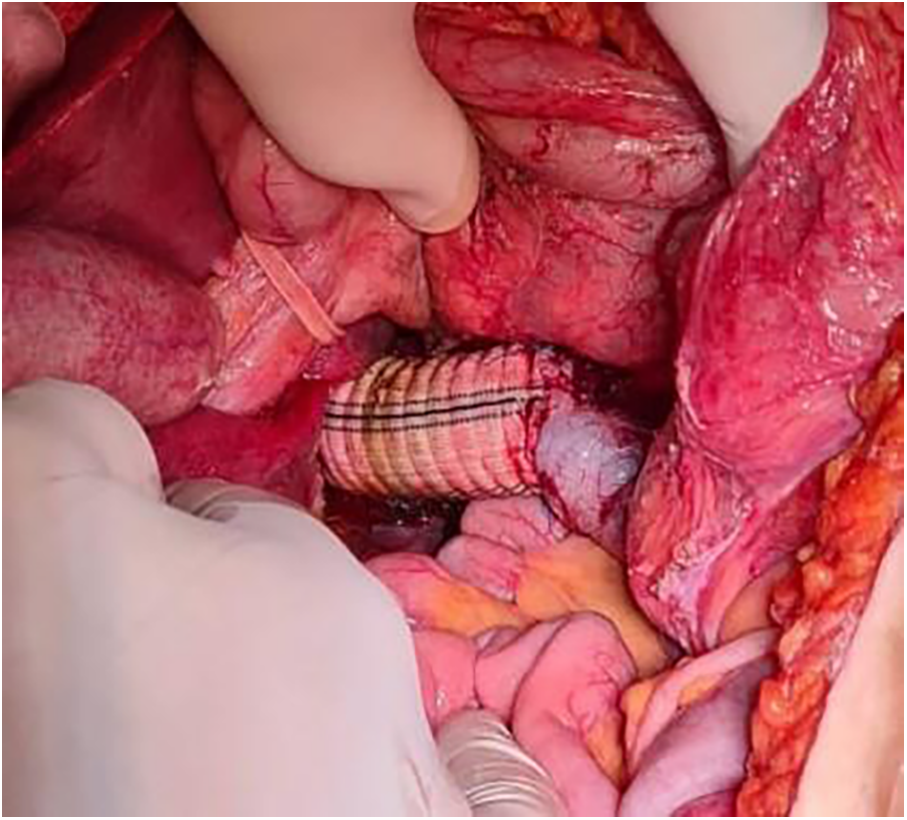
IVC graft, intraoperative image of IVC graft placement.

Urine output was checked after 15 min and showed 15 cc of clear urine. Intraoperative blood loss was approximately 3 L, and required 6 L of crystalloids, 7 packs of packed red blood cells, 6 packs of fresh frozen plasma and platelets, and 4 packs of cryoprecipitate. The surgery lasted for approximately 7 h, and there were no intraoperative complications (Figure 2—IVC Graft).

Histopathology of the specimen showed a 9 cm leiomyosarcoma pushing into the renal sinus and ipsilateral adrenal gland, with a negative ureteric margin. The mass was superiorly limited by a thin fibrous capsule. Separated wall pieces with chronic inflammation and focal mural thrombosis were negative. The margins were negative all around. Immunohistochemistry revealed that the tumor cells were positive for SMA and desmin.

Postoperatively, the patient was kept for 48 h in the intensive care unit (ICU) for close observation and then transferred to the general ward. In the following hours, she developed intra-abdominal bleeding with hematoma formation and bloody drain output, managed conservatively with blood product transfusion. She was discharged 2 weeks later in good general condition. A week after discharge, she complained of abdominal pain and distention. Imaging revealed intraperitoneal fluid accumulation. An ultrasound-guided drain was inserted, draining old blood and successfully resolving her symptoms.

A 2-month CT scan follow-up showed only postoperative changes, with no evidence of metastatic disease or local recurrence. The patient was followed up closely for 4 months after surgery, and her condition showed an uneventful course. She was sent to an oncological center and started on a radiotherapy course.

### 2.2 Case 2

A 75-year-old female patient was referred to our hospital for an excisional biopsy of a retroperitoneal mass. She had a past medical history of diabetes mellitus and hypertension. She previously underwent bilateral knee replacement and hip replacement surgery. The patient's history dates back to 2016 when she noticed a lump on the front of her head. Following medical consultation, the mass was excised but recurred thrice in 2016, 2020, and 2021.

Histopathological examination following its third recurrence revealed squamous cell carcinoma. A positron emission tomography (PET) scan following oncological referral revealed various masses, including a mildly hypermetabolic heterogeneous fat-containing mass lesion in the right retroperitoneal region, pushing the right kidney antero-inferiorly, measuring approximately 10 cm in the longest axial diameter with fluorodeoxyglucose (FDG) uptake up to 3, with a similar 1.8 mass in the left upper quadrant of the mesentery, although with insignificant FDG uptake. There was also a hypermetabolic nodule in the right adrenal gland measuring 2.2 cm axially with a maximum standardized uptake value of up to 3.9.

On abdominopelvic CT, there was a large heterogeneous fat containing a retroperitoneal-retrorenal relatively well-defined mass measuring 11 × 9.5 × 7.5 cm.

The mass showed fat haziness and increased vascularity, mainly arising from the right renal artery while abutting the whole upper posterior renal surface without definite invasion. Another small heterogeneous focal lesion measuring 1.7 cm was also observed, anterior to the lower pole of the left kidney, and two right adrenal gland nodules measuring 2.4 cm and 1 cm were suspicious of pathological activity.

Following high suspicion of liposarcoma, considering the typical presentation of recurrent malignancies, and multiple resections, the patient was referred to our department for an excisional biopsy. Upon admission and review of her reports, it was decided to perform a laparotomy with retro-peritoneal mass excision and right adrenalectomy. She had no complaints at this point.

Upon examination, she had no palpable abdominal masses other than a small umbilical hernia. Preoperatively, she was prepared for surgery and had no abnormalities on the echocardiogram. She underwent resection of a right-sided retroperitoneal mass with a right nephrectomy. Intraoperatively, a large right-sided retroperitoneal mass, approximately 10 × 7 cm, was found invading the upper pole of the right kidney, which was completely resected with no immediate complications.

The resected mass was sent for histopathological examination. It consisted of kidney tissue and a hemorrhagic necrotic mass at the upper pole measuring 7 × 5 × 5 cm and was surrounded by a thin capsule. Upon opening, the kidney parenchyma was almost unremarkable, with mild chronic interstitial nephritis, and the vessel margins were negative. The viable areas of the mass showed a white and yellow cut surface. The histopathological examination of the mass showed a well-differentiated liposarcoma. Perinephric fat measuring 2 cm in thickness is identified. Unremarkable adrenal gland tissue was also present. An incidental adrenal mass measuring 2.5 × 1.5 × 1 cm was also identified as an adrenocortical adenoma.

Following surgery, after recovery with stable vital signs, the patient was transferred to the ICU for 24 h for close observation. Postoperatively, renal ultrasound was performed and showed no abnormalities. However, the patient's creatinine was rising, and acute kidney injury was suspected. Thus, she was maintained on IV fluids and closely monitored by the nephrology team. They reported steady improvement and normalization over the next 3 days.

Prior to discharge, the patient was well and had her drain removed. She had no further complications. She was then referred to a specialist oncology center for further management, where she did not receive adjuvant chemotherapy. To date, the tumor has not recurred, despite high rates of recurrence concerning retroperitoneal liposarcomas.

### 2.3 Case 3

A 59-year-old male patient presented to our department complaining of a lump on the left side of his abdomen he had noticed a year and a half beforehand. The patient had a history of diabetes mellitus for 25 years, ischemic heart disease, and a COVID-19 infection a month and a half ago. He previously underwent 4 cardiac catheterizations with stenting, the last of which was a year ago. He also underwent an open appendectomy 25 years ago.

The patient had initially noticed the mass by accident but neglected it, as it had no associated symptoms. Since then, the lump has grown, causing the patient to develop constipation 6 months ago and preventing him from passing stool more than once every 4 days. The patient also mentioned that he had recently become anorexic and lost 3 kg in the past 3 months. The culmination of these symptoms prompted him to seek medical attention 20 days ago.

Thus, he underwent an abdominal ultrasound, which revealed a well-defined large heterogeneously enhanced soft tissue mass with few calcific foci in the left side of the abdomen, located antero-inferiorly to the left kidney measuring approximately 16 × 14 × 11 cm.

**Figure 3:**
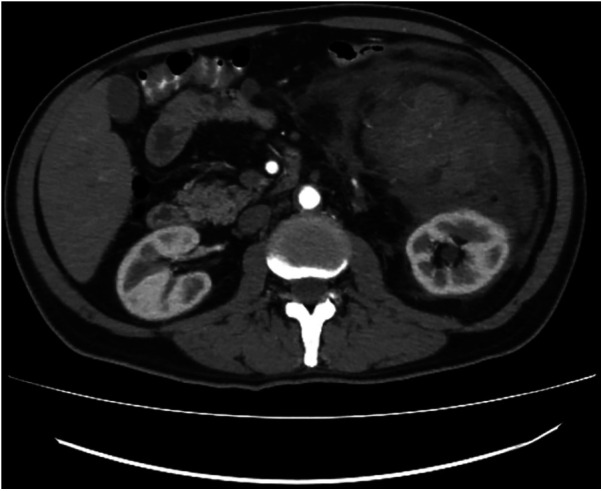
Ct angio, CT findings for case 2.

The seemingly mesenteric mass was surrounded by mesenteric fat stranding, a mild amount of free fluids, and loculated fluid superior to the mass, which measured 8 × 12.5 × 9.5 cm. Although there was no evidence of invasion of surrounding structures, it was visualized compressing the transverse and descending colon. Multiple mildly enlarged para-aortic lymph nodes, the largest measuring 2.2 × 1.2 cm on the left side (Figure 3—CT Angio).

The right kidney showed mild hydronephrosis with perinephric free fluid and fat stranding. The liver and spleen were also enlarged. Furthermore, the urinary bladder wall was thickened with a visible urachus.

On examination, his abdomen was distended with full flanks and a palpable, irregular, slightly tender left-sided abdominal mass of approximately 10 × 15 cm extending from the left costal margin to the left iliac crest. He also had a visible appendectomy scar at the McBurney point. Thus, the patient was admitted to excise the mass.

A multidisciplinary team of pulmonologists, cardiologists, and urologists provided their recommendations following spirometry, abdominal CT angiography, and echocardiography.

Pulmonary function tests showed no abnormalities. CT findings suggest post-COVID changes, although PCR testing for COVID-19 was negative. Echocardiography findings were significant for mild diastolic dysfunction and pleural effusion. Thus, the patient was started on antiplatelet therapy and high doses of statins. Upon urologic recommendations, a double J stent was used, and the procedure was performed via a retroperitoneal approach.

The patient thus underwent midline laparotomy and layer dissection to afford access to the peritoneal cavity. We attempted to bypass the peritoneum from the left side, however we were unable to. Subsequently, we dissected the peritoneum. A huge mass was apparent in the retroperitoneum attached to the spleen, distal pancreas, splenic flexure, and engulfing the left kidney and ureter. The mass was severely adherent to surrounding structures. There was also an enlarged para-aortic and mesenteric lymph node.

We began to carefully dissect the peritoneal covering of the mass starting from the inferolateral border. We attempted medial dissection to separate the mass from the splenic flexure, however we were unsuccessful.

We were able to afford access to the inferior border of the mass and continued dissection from there. We resected the splenophrenic ligament and managed to free the spleen laterally. Since the distal pancreas was adherent to the mass, we had to maneuver with careful dissection and suction, to identify the splenic vein and artery. We ligated the splenic artery and dissected the distal pancreas and splenic vein. We then resected the descending colon and splenic flexure.

We identified, ligated, and cut the renal artery, vein, and ureter, and then proceeded to do *en bloc* nephrectomy. Despite being adherent to the iliopsoas muscle, the mass was separated from it with fine dissection. We performed para-aortic lymphadenectomy extending to lymph nodes surrounding the left common iliac artery. The sigmoid mesentery and enlarged mesenteric lymph node were hard, and so were included in the resection under high suspicion of involvement. We concluded with colo-colic anastomosis and securing hemostasis.

The patient then received 2 units of packed red blood cells. He also had drains, a nasogastric tube, and a Foley catheter.

On histopathological examination, the specimen consisted of a large mass measuring 25 × 20 × 18 cm attached to multiple viscera. Sectioning of the mass showed a mucoid and gray cut surface attached to the mass, and the left kidney measured 9 × 4 × 2 cm. The colon part measured 12 × 5 cm, the splenic part measured 12 × 6 × 3 cm, and the pancreatic tail measured 7 × 7 × 3 cm. The neoplastic margin was circumferentially 2 mm away from the margins, including the mesentery. The extent of necrosis extended to up to 20%.

The mass was determined to be a dedifferentiated liposarcoma, with a high mitotic rate and necrotic tissue. The mass was invading adjacent structures, including the colon, kidney, pancreas, and hilum of the spleen, along with three reactive lymph nodes. The tumor cells showed focal positivity for S100 and MDM2 markers on immunohistochemistry.

Postoperatively, he was transferred to the ICU for close observation. His Foley catheter was changed as he was anuric, and he was administered furosemide, after which his urine output improved. He was also started on antibiotic therapy. He also developed sudden onset dysarthria, difficulty speaking, and transient left upper and lower limb weakness for 1 min. There were, however, no abnormalities on brain CT.

During his second postoperative day, he developed acute kidney injury and was oliguric but was successfully managed with furosemide. All catheter lines and the NGT tube were removed upon leaving the ICU. His Foley catheter was removed 1 day later. The patient then developed hypertension, which was started on aminocaproic acid and kept on carvedilol. However, his hypertension persisted. Thus, he was successfully managed with nifedipine, and previous medications were stopped.

The patient complained of dyspnea on mild exertion but showed no new abnormalities on echocardiography. Electrocardiography showed no acute changes. Chest CT revealed moderate right-sided pleural effusion. By the evening, the patient's symptoms had resolved, and his left drain was removed. His right drain was removed 1 day later, and the patient was discharged in good condition.

## 3 Discussion

Among retroperitoneal sarcomas, well-differentiated liposarcomas are the most commonly presented, followed by dedifferentiated liposarcomas and leiomyosarcomas (1, 2, 5, 6). Both types of liposarcomas show amplification of MDM2, while leiomyosarcomas are negative for S100 and express desmin (5). Retroperitoneal sarcomas and liposarcomas, in particular, do not show symptoms until they grow to massive sizes, through which they compress surrounding structures that generate a variety of vague neurological, musculoskeletal, gastrointestinal, and urinary symptoms. In most cases, they remain asymptomatic. These symptoms are commonly thought to be the result of other less serious pathologies, which further delay proper management (6, 7).

In retroperitoneal sarcoma cases, diagnosis and treatment are of utmost importance and intricacy. Due to their rarity and the vagueness of their presentation, in concomitance with their poor prognosis, it is crucial to identify leading trends concerning their diagnosis and appearance on imaging modalities. Especially in the case of retroperitoneal liposarcoma, delayed imaging may lead to a large unresectable tumor, inevitably worsening outcomes (1, 3, 7, 9). Other neoplasms may mimic the presentation of retroperitoneal sarcomas, thus demanding definitive differentiation, especially in the face of different treatment protocols (7, 9).

Despite the efficacy of percutaneous biopsy, it is not always possible due to difficulty in attaining the specimen. Thus, CT offers an excellent solution for first-line imaging. A mixture of MRI and CT is beneficial in a mutually compensatory manner, especially when CT is not entirely conclusive (1, 5, 6, 7, 9). PET scans, although not routinely used, play a pivotal role in problem-solving or incidental findings, as in our second case (1). Although biopsy offers a definitive diagnosis, it is still important to act promptly under high suspicion of retroperitoneal sarcomas, as in 2 of our cases. Furthermore, liposarcomas predominate the fat tissue masses in the retroperitoneum, which guides management upon detection (6, 9).

It is also essential to consider the implications of undetermined extensions of masses obscured by viscera, especially in liposarcomas. This plays a significant role in determining the surgical approach and decreasing complications (1, 5).

The mainstay of treatment is surgical resection. Previous studies have shown that R0 resection reduces abdominal recurrence to approximately 10% in contrast to 50% in R1, which is the main predictor of mortality in retroperitoneal sarcoma. Multidisciplinary involvement is also a vital determinant of outcomes (1, 2, 5, 8, 9).

Extra care must be placed into the tumor's extension into multiple organ systems. Multiple disciplines inevitably involve renal involvement, alimentary invasion, or encirclement of the vasculature. In the case of resected organs, which account for 75% of cases, the importance of this multidisciplinary approach reaches its peak (1, 5, 8, 9).

Possible paths to resection include wide local excision, compartmental, and complete multiorgan evisceration, with emphasis on removal from the first try, to prevent further seeding and recurrence. Compartmental resection, which involves the removal of unaffected soft tissue within the vicinity of the tumor, was shown to be marginally more effective at reducing recurrence, while acceptably compromising morbidity. This plays into the risk of non-apparent infiltration, especially in cases where invasion is likely, including vascular supply, or organ encasement, adjacency, or adherence to the tumor. It is important to acknowledge the removal of major vessels or nerves only if the tumor involves them as well. Palliative and distant metastasis options are available though somewhat controversial (10).

Despite trends in R0 resection, recurrence is still a problem. According to some accounts, it may even approach 85%, involving both local and distant recurrence. Certain low-risk recurrences may be followed up, or alternatively undergo salvage surgery, though it is also debatable due to the risk of a scarred abdomen. The latency in deciding to operate is also controversial as the risk of recurrence grows beyond 3 times, but also enables the finding of distant or alternative foci of recurrence (10).

It is especially important to take into account leiomyosarcomas involving or arising from major vessels, the chief of which is IVC (9). Not only does it account for 0.5% of retroperitoneal sarcomas, but the involvement of the vessel may be complicated with further extension of the tumor or compression, influencing morbidity and presentation (9, 10). Under the decision of vascular resection is a background of risk vs. reward. Despite limited studies concerning Dacron grafting and venous reconstruction in retroperitoneal sarcomas, the outlook is promising as a readily available method of IVC reconstruction, especially in low-resource settings, considering the difficulty of procuring donor parts (11). Though vascular resection plays an important role in R0 resection, it carries significant risk and high morbidity rates, irrespective of reconstruction modality. Despite this, most complications can be dealt with, with relatively low risk. This makes them effective and relatively safe options to achieve R0 resection (11).

Several points contribute to the ultimate surgical plan. How much of the vessel should be removed, is it an artery or a vein, and what should it be replaced with? In the case of the aorta, the sheer amount of tissue that needs to be resected in such a vital place rules out the procedure. Usually, prostheses are reserved for arterial grafts. In the case of superior mesenteric involvement, the tumor is likely unresectable, though there are reports of select cases in which segmental resection and prosthesis placement are a viable option (11, 12).

In the case of IVC involvement, tumor site, size, adequate collaterals, and lumen patency are the most important deciding factors. Several options are available. Primary repair is useful in cases of partial resection where lumen patency may be compromised to less than 50%. In greater resections, or when ligation is not an option, grafts are more useful, though also come at the cost of lifelong anticoagulation and greater post-operative complications. One problem with biological grafts such as aortic grafts is that they are in short supply in-low income areas, such as our case. Should the lumen be obliterated, IVC ligation is viable if sufficient collaterals are available. In all cases of IVC involvement, renal vascular intricacy is advised, especially when preserving the right kidney, as it lacks sufficient collaterals and must therefore be re-implanted (11, 12).

Although evidence shows that renal artery embolization before radical nephrectomy for renal masses seems to be a valuable tool in the surgical management of a large mass and advanced disease, as it induces preoperative infarction and facilitates surgical intervention (13), this was not possible in our first case, as radiological evaluation did not confirm the renal vs. hepatic origin of the tumor. There was no single specific feeding vessel.

## 4 Conclusion

Invasive retroperitoneal sarcomas of different histological types and clinical stages represent a technical challenge. Careful preoperative investigation and an experienced, dedicated multidisciplinary team of surgeons from related fields, including vascular, urologic, and hepatobiliary surgeons, are usually needed for a safe and successful R0 resection despite extensive tumor involvement.

## Data Availability

The original contributions presented in the study are included in the article, further inquiries can be directed to the corresponding author.
